# Emerging Therapies in IgA Nephropathy: From A Proliferation-Inducing Ligand (APRIL) and B-cell Activating Factor (BAFF) Inhibitors to Precision Medicine

**DOI:** 10.7759/cureus.99870

**Published:** 2025-12-22

**Authors:** Ishwor Sharma, Raju Panta

**Affiliations:** 1 Nephrology, Nephrology Associates of Kentuckiana, Louisville, USA; 2 Physiology and Pathology, Burrell College of Osteopathic Medicine, Melbourne, USA

**Keywords:** april, baff, end stage kidney disease (eskd), galactose-deficient iga1, iga nephropathy (igan), immunomodulators, iptacopan, kdigo, sglt-2 inhibitor, sparsentan

## Abstract

IgA nephropathy (IgAN) is the most prevalent primary glomerular disease worldwide and a significant contributor to end-stage kidney disease (ESKD). Traditional management has centered on supportive care and non-specific immunosuppression, but recent advances in the understanding of pathogenic pathways have catalyzed the development of targeted therapies. This review synthesizes current evidence on evolving treatments, with a focus on A Proliferation-Inducing Ligand (APRIL) and B-cell Activating Factor (BAFF) (e.g., sibeprenlimab, atacicept, povetacicept, telitacicept), complement pathway modulators (e.g., iptacopan, cemdisiran, ravulizumab), and novel agents such as felzartamab and sparsentan. It also explores precision medicine strategies, including biomarker-guided therapy, individualized risk stratification, and combination regimens. Supported by high-quality recent clinical trial data and the latest kidney disease outcome guidelines, these innovations represent a paradigm shift toward personalized, disease-modifying treatment in IgAN, offering a new horizon for improved renal outcomes and long-term disease control.

## Introduction and background

IgA nephropathy (IgAN) is defined histologically by the presence of dominant or codominant IgA-containing immune complex deposits in the glomeruli, often accompanied by varying degrees of inflammation and/or sclerosis [1]. IgAN has a highly variable clinical spectrum ranging from asymptomatic urinary abnormalities like microscopic hematuria and/or proteinuria to severe presentations like rapidly progressive glomerulonephritis or nephrotic syndrome. The most common presentations are synpharyngitic macroscopic hematuria (often after upper respiratory or gastrointestinal infections) and persistent microscopic hematuria and/or proteinuria detected on routine screening. IgAN is the most common primary glomerulonephritis worldwide and a leading cause of kidney failure in both children and adults. Its global incidence is estimated to be at least 2.5 cases per 100,000 population [2]. 

IgAN shows marked differences in prevalence across regions. In East Asia, particularly Japan and China, IgAN accounts for 40-50% of all biopsy-proven glomerulonephritis, whereas in Europe and North America, it represents 20-30% [3]. A male predominance is observed in Western populations (male-to-female ratio is approximately 2-3:1), while in East Asia, the distribution is closer to equal. The 10-year risk of halving estimated glomerular filtration rate (eGFR) or progressing to end-stage kidney disease (ESKD) is approximately 26%, highlighting the variability in disease course [3,4]. 

Current management of IgAN is largely supportive, with few disease-specific treatments available. Recently, multiple clinical trials have explored novel therapies targeting the underlying disease mechanisms. These advances may soon transform the standard of care, making it important to review and understand these emerging options.

Methodology

This review was conducted to synthesize current evidence on emerging therapeutic strategies in IgAN, with a focus on A Proliferation-Inducing Ligand (APRIL)/B-cell Activating Factor (BAFF) inhibitors, complement-targeted agents, and approaches to precision medicine. A comprehensive literature search was performed using PubMed, Scopus, and Google Scholar databases for articles published between January 2005 and November 2025. Search keywords and medical subject headings included IgA nephropathy, APRIL, BAFF, sibeprenlimab, atacicept, povetacicept, telitacicept, complement inhibition, iptacopan, cemdisiran, ravulizumab, precision medicine, and Kidney Disease: Improving Global Outcomes (KDIGO) guidelines.

Clinical trial registries (ClinicalTrials.gov and EU Clinical Trials Register) were reviewed to identify ongoing and completed trials relevant to IgAN. Inclusion criteria encompassed peer-reviewed original research articles, clinical trial reports, guideline publications, and expert reviews focused on therapeutic efficacy, safety, and mechanistic insights. Non-English articles, case reports, and studies lacking clinical relevance were excluded.

Data were extracted on study design, patient population, intervention type, outcomes (e.g., proteinuria reduction, eGFR stabilization), and safety profiles. Emphasis was placed on phase 2 and 3 trials, KDIGO 2025 recommendations, and biomarker-driven strategies. The review was structured to highlight mechanistic rationale, clinical efficacy, and future directions in personalized IgAN care.

## Review

Pathogenesis of IgAN

IgAN is characterized by mesangial deposition of galactose-deficient IgA1 (Gd-IgA1) and immune complex formation, leading to glomerular inflammation and fibrosis [4]. The “four-hit hypothesis” outlines its pathogenesis: 

Hit 1: Overproduction of Gd-IgA1, Primarily From Mucosal Plasma Cells

Gd-IgA1, which lacks terminal galactose in its hinge region, is mainly produced in the gut and nasal mucosa. Genetics can influence this abnormal glycosylation, but it is not solely responsible for disease development. Cytokines such as APRIL and BAFF promote the survival of mucosal B cells and the formation of plasma cells that secrete Gd-IgA1, contributing to the pathogenesis of IgA nephropathy [2,5-12].

Hit 2: Formation of Anti-Gd-IgA1 Autoantibodies

Gd-IgA1 exposes terminal N-acetylgalactosamine (GalNAc) residues in the hinge region of the IgA1 molecule. These exposed residues are normally concealed by O-linked glycans, so their presence creates a neoepitope that the immune system recognizes as foreign. IgG and IgA autoantibodies are generated against these neoepitopes. The IgG subclass is particularly implicated in forming pathogenic immune complexes, though IgA autoantibodies also contribute. These antibodies bind to Gd-IgA1, forming circulating immune complexes that are prone to deposition in the glomerular mesangium [2,3,9-12].

Hit 3: Immune Complex Formation and Mesangial Deposition

Once anti-Gd-IgA1 autoantibodies (IgG and IgA) bind to Gd-IgA1, they form large circulating immune complexes. These complexes are structurally abnormal, with increased molecular size and altered charge, making them resistant to normal hepatic clearance mechanisms. The immune complexes remain in the bloodstream for prolonged periods. Their persistence is facilitated by impaired clearance via the asialoglycoprotein receptor in the liver, which normally removes glycosylated IgA1. This allows pathogenic complexes to accumulate and eventually deposit in the glomerular mesangium [2,3,9-12].

Hit 4: Glomerular Inflammation and Injury

Once immune complexes are deposited in the mesangium, mesangial cells internalize these complexes and become activated. Activated mesangial cells proliferate and secret pro-inflammatory cytokines such as interleukin-6 (IL-6) and tumor necrosis factor-alpha (TNF-α), growth factors such as transforming growth factor-beta (TGF-β) and platelet-derived growth factor (PDGF), and chemokines that recruit immune cells. This leads to mesangial matrix expansion, glomerular injury, and progressive scarring. [2,3,9-13].

Complement-mediated damage in IgAN represents a central effector mechanism linking immune complex deposition to progressive glomerular injury. Once Gd-IgA1-containing immune complexes are deposited in the mesangium, they activate the complement system, particularly through the alternative and lectin pathways. This activation generates potent inflammatory mediators such as the anaphylatoxins C3a and C5a, which serve as chemoattractants for neutrophils and macrophages, thereby amplifying local inflammation within the glomerulus. In addition, complement activation leads to assembly of the membrane attack complex (MAC, C5b-9), which directly damages glomerular endothelial and mesangial cells, contributing to structural injury and loss of renal function. Histopathological studies consistently demonstrate complement deposition, particularly C3, C4d, and mannose-binding lectin (MBL), in renal biopsies of affected patients, and the extent of such deposition correlates strongly with disease severity and progression. This evidence underscores the pivotal role of complement-mediated injury in the pathogenesis of IgAN and highlights complement components as both biomarkers of disease activity and potential therapeutic targets [9,12-14].

Up to 50% of patients progress to ESKD within 20 years [15]. APRIL and BAFF are cytokines that promote B-cell maturation, plasma cell survival, and IgA class switching. APRIL is a member of the TNF superfamily. It plays a key role in IgA-producing plasma cells and the production of Gd-IgA1, which is central to the pathogenesis of IgA nephropathy. BAFF, also known as B-lymphocyte stimulator (BLyS), is another TNF family cytokine that promotes B-cell maturation, differentiation, and immunoglobulin production. Elevated BAFF levels are associated with autoimmune diseases and are implicated in the overproduction of pathogenic IgA in IgAN. Elevated serum levels of both are observed in IgAN and correlate with disease severity, for example, higher proteinuria and lower eGFR [16,17].

APRIL and BAFF inhibitors in IgAN

Sibeprenlimab

Sibeprenlimab, a fully humanized IgG2 monoclonal antibody, represents a novel therapeutic approach in IgAN by selectively neutralizing APRIL while sparing BAFF, thereby avoiding broad B-cell depletion. This targeted mechanism is designed to reduce the production of Gd-IgA1, a key pathogenic driver of IgAN. In the phase 2b ENVISION trial, sibeprenlimab demonstrated robust, dose-dependent efficacy, achieving up to a 65% reduction in circulating Gd-IgA1 levels and a 47-62% reduction in proteinuria compared with placebo, without compromising eGFR or vaccine responsiveness. These findings highlight preserved immune competence during therapy, an important distinction from broader immunosuppressive strategies. More recently, interim results from the ongoing phase 3 VISIONARY trial confirmed the clinical promise of sibeprenlimab, showing a placebo-adjusted 51.2% reduction in proteinuria at nine months and a favorable safety profile, with lower rates of serious adverse events compared to placebo. Collectively, these data support sibeprenlimab as a disease-modifying therapy for patients with IgAN who remain at risk of progression despite optimized supportive care, positioning APRIL inhibition as a key therapeutic strategy in the evolving treatment landscape [4,18].

Atacicept

Atacicept is a recombinant fusion protein designed to simultaneously neutralize both BAFF and APRIL, thereby targeting two critical cytokines involved in the pathogenesis of IgAN. By inhibiting both pathways, atacicept aims to reduce the production of Gd-IgA1 and limit immune complex formation more effectively than single-cytokine blockade. In the ORIGIN 3 phase 3 trial, weekly administration of atacicept 150 mg resulted in a 45.7% reduction in proteinuria from baseline and a 41.8% reduction compared with placebo at 36 weeks (p<0.0001), demonstrating clinically meaningful efficacy. Furthermore, atacicept achieved a 68% reduction in circulating Gd-IgA1 levels and an 81% resolution of hematuria, underscoring its impact on key pathogenic drivers of IgAN. Importantly, the therapy was well tolerated, with fewer serious adverse events than placebo (0.5% vs 5.1%) and no evidence of opportunistic infections or hypogammaglobulinemia, highlighting its favorable safety profile [16,19]. The dual inhibition strategy is thought to provide more comprehensive and durable disease control by preventing compensatory upregulation of either BAFF or APRIL, positioning atacicept as a promising disease-modifying therapy in patients with persistent proteinuria despite optimized supportive care [20,21].

Povetacicept

Povetacicept is an investigational fusion protein that simultaneously inhibits both BAFF and APRIL, two cytokines that play complementary roles in B-cell survival, maturation, and immunoglobulin production. By targeting both pathways, povetacicept aims to suppress the generation of Gd-IgA1, reduce autoantibody formation, and limit pathogenic immune complex deposition in the glomerular mesangium. This dual inhibition strategy is thought to provide more comprehensive disease control than selective blockade, as it prevents compensatory upregulation of either BAFF or APRIL signaling [20,21]. 

In the RUBY-3 phase 1/2 trial, povetacicept, administered subcutaneously every four weeks, demonstrated robust efficacy over 48 weeks, with a 64% reduction in urinary protein-to-creatinine ratio (UPCR) and a 77% decline in circulating Gd-IgA1 levels, highlighting its ability to directly modulate key pathogenic drivers of IgAN. Importantly, among patients receiving the 80 mg dose, 53% achieved clinical remission, defined as UPCR <0.5 g/g, minimal hematuria, and <25% decline in eGFR. This remission rate is notable given the refractory nature of IgAN in patients with persistent proteinuria despite optimized supportive care [21].

The safety profile of povetacicept has been favorable, with no serious adverse events attributed to the drug and no evidence of opportunistic infections, immunosuppression, or hypogammaglobulinemia. Preservation of normal immunoglobulin levels and vaccine responsiveness suggests that povetacicept achieves targeted immunomodulation without compromising systemic immune competence, which is a critical advantage over traditional immunosuppressive therapies. Beyond IgAN, povetacicept’s mechanism of dual BAFF/APRIL inhibition positions it as a potential best-in-class therapy for other autoimmune glomerulopathies, such as lupus nephritis and membranous nephropathy, where dysregulated B-cell activity and abnormal immunoglobulin production contribute to disease progression. The RUBY-3 findings support further phase 3 evaluation and highlight povetacicept’s promise as a disease-modifying agent capable of altering the natural history of IgAN by reducing proteinuria, suppressing Gd-IgA1, and promoting durable remission [19-21].

Telitacicept

Telitacicept is a recombinant fusion protein that simultaneously neutralizes BAFF and APRIL, aiming to dampen B-cell survival signals and upstream drivers of pathogenic IgA production in IgAN. In a large, multicenter, randomized, double-blind, placebo-controlled phase 3 trial in China involving 318 adults with high-risk IgAN on stable background therapy, once-weekly subcutaneous telitacicept 240 mg met its primary endpoint, producing a 55% reduction in 24-hour UPCR at 39 weeks versus placebo (p<0.0001); a separate report quantified the reduction as 58.9% on telitacicept versus 8.8% on placebo and detailed proteinuria thresholds achieved, underscoring the depth of effect on this validated surrogate marker [22-24]. Kidney function outcomes favored telitacicept, with a lower proportion of patients experiencing a ≥30% decline in eGFR (6.3% vs 27.0%) and signals of eGFR stabilization over the 39-week period, supporting disease-modifying potential beyond proteinuria reduction [25]. Safety was favorable, with fewer serious adverse events than placebo (2.5% vs 8.2%) and a profile characterized by predominantly mild-to-moderate treatment-emergent events, aligning with the strategy’s targeted immunomodulation rather than broad immunosuppression [24,25]. Collectively, these findings position dual BAFF/APRIL inhibition with telitacicept as a compelling approach to achieve deeper proteinuria reductions, preserve kidney function, and potentially deliver more durable control by preventing compensatory pathway upregulation [22-25].

Complement inhibitors in IgAN

Iptacopan

Iptacopan is a first-in-class, selective oral inhibitor of factor B, a central component of the alternative complement pathway, which plays a pivotal role in the amplification of complement activation in IgAN and C3 glomerulopathy (C3G). By specifically targeting factor B, iptacopan interrupts the amplification loop of the alternative pathway while sparing the classical and lectin pathways, thereby offering a more tailored approach to immune modulation that avoids broad complement blockade. This mechanism is particularly relevant in complement-driven glomerulopathies, where dysregulated alternative pathway activity contributes to mesangial deposition, inflammation, and progressive renal injury [14,20,26,27].

Clinical evaluation has demonstrated encouraging results. In the APPEAR-C3G phase 2 trial, iptacopan showed a favorable safety profile and provided mechanistic proof-of-concept for complement inhibition in rare glomerular diseases, with reductions in complement activation markers and stabilization of kidney function [26]. More recently, the APPLAUSE-IgAN phase 3 trial confirmed its therapeutic potential in IgAN, with iptacopan achieving a mean reduction in UPCR of 38.3% at nine months compared to placebo (95%CI, 26.0-48.6; p<0.001), alongside stabilization of eGFR. Importantly, treatment was well tolerated, with no excess risk of serious infections or hypogammaglobulinemia, underscoring the advantage of selective pathway inhibition [28]. Collectively, these findings support iptacopan as a promising disease-modifying therapy for complement-mediated glomerulopathies, particularly in patients at risk of rapid progression despite optimized supportive care.

Cemdisiran 

Cemdisiran is an investigational small-interfering ribonucleic acid (siRNA) therapeutic that selectively targets hepatic synthesis of complement component C5, thereby attenuating downstream complement-mediated inflammation implicated in the pathogenesis of IgAN. By reducing circulating C5, cemdisiran aims to limit the generation of C5a anaphylatoxin and the membrane attack complex (C5b-9), both of which contribute to glomerular injury [14,20]. 

In a multicenter, randomized, double-blind, placebo-controlled phase 2 trial, Barratt et al. evaluated cemdisiran in adults with biopsy-confirmed IgAN and persistent proteinuria despite optimized supportive care. Treatment with cemdisiran achieved a mean 98.7% reduction in serum C5 levels at week 32, confirming potent target engagement. Although the primary endpoint of proteinuria reduction did not reach statistical significance, a trend toward decreased proteinuria over 12 months was observed, suggesting potential clinical benefit in subsets of patients with higher complement activation. Importantly, cemdisiran was well tolerated, with no serious adverse events attributed to the drug, and no evidence of opportunistic infections or hypogammaglobulinemia, underscoring its favorable safety profile [29]. These findings support further investigation of cemdisiran as a complement-targeted therapy in IgAN, particularly in combination with other agents or in populations enriched for complement-driven disease activity.

Ravulizumab

Ravulizumab is a long-acting monoclonal antibody that selectively inhibits complement component C5, thereby preventing cleavage into C5a and C5b and subsequent formation of the membrane attack complex (MAC, C5b-9). By blocking terminal complement activation, ravulizumab mitigates glomerular inflammation and injury while preserving upstream immune defense mechanisms mediated by the classical and lectin pathways. This targeted approach is particularly relevant in IgAN, where complement dysregulation contributes to mesangial deposition, proteinuria, and progressive renal decline [14,20]. In the phase 2 SANCTUARY trial, Lafayette et al. demonstrated that ravulizumab significantly reduced proteinuria and stabilized kidney function compared with placebo over a 12‑month treatment period, with a favorable safety profile and no treatment-related serious adverse events. These findings underscore the therapeutic potential of terminal complement inhibition in IgAN [30]. As emphasized by Lim et al., complement-targeted therapies such as ravulizumab represent a paradigm shift in IgAN management, offering disease-modifying potential beyond traditional immunosuppression and supportive care [20].

Other emerging therapies in IgAN

Felzartamab

Felzartamab is a human monoclonal antibody directed against CD38, a surface antigen expressed on plasma cells, offering a novel immunomodulatory strategy in IgAN. By selectively depleting CD38-positive plasma cells, felzartamab reduces the production of aberrant Gd-IgA1, a central driver of immune complex formation and mesangial deposition in IgAN [20]. In a phase 2a randomized, double-blind, placebo-controlled trial, Floege et al. reported that felzartamab achieved a significant reduction in proteinuria and stabilization of kidney function over 12 months, with a favorable safety profile and no unexpected adverse events, underscoring its tolerability in patients with persistent proteinuria despite optimized supportive care. The mechanism of action, targeted plasma cell depletion, distinguishes felzartamab from broader immunosuppressive therapies by focusing on the pathogenic source of abnormal IgA1 rather than global B-cell suppression [31].

Further evidence from the IGNAZ study, presented by Barratt et al., reinforced felzartamab’s potential as a disease-modifying therapy. In this study, felzartamab demonstrated sustained reductions in proteinuria and complement activation markers, highlighting its ability to modulate both upstream immunoglobulin production and downstream inflammatory pathways [32]. Together, these findings suggest that felzartamab may provide durable disease control in IgAN by directly targeting the plasma cell compartment responsible for pathogenic IgA1 synthesis, thereby addressing the root cause of disease progression.

*Sparsentan* 

Sparsentan is a novel dual endothelin type A (ETA) and angiotensin II type 1 (ATII-1) receptor antagonist that has emerged as a first-in-class therapy for IgAN. By simultaneously blocking two key pathways implicated in glomerular injury, endothelin-mediated vasoconstriction and angiotensin II-driven intraglomerular hypertension, sparsentan provides a synergistic renoprotective effect beyond conventional renin-angiotensin system (RAS) inhibition [14,20]. In the PROTECT phase 3 trial, sparsentan demonstrated sustained efficacy over two years, achieving a 38.3% mean reduction in UPCR at week 110 compared with irbesartan (p<0.0001). Importantly, sparsentan was associated with a slower decline in eGFR, suggesting disease-modifying potential beyond proteinuria reduction. Interim analyses confirmed early and consistent reductions in proteinuria by week 36, reinforcing its rapid onset of action and durable efficacy. The safety profile was favorable, with no excess of serious adverse events compared to irbesartan, underscoring its tolerability in long-term use [33,34].

Reflecting these findings, the KDIGO 2025 Clinical Practice Guideline for Glomerular Diseases recommends sparsentan as a first-in-class, disease-modifying agent in IgAN, highlighting its ability to deliver sustained proteinuria reduction, preserve kidney function, and provide a favorable safety profile [16]. Collectively, sparsentan represents a paradigm shift in IgAN management, offering targeted dual-pathway blockade that addresses both hemodynamic and inflammatory drivers of disease progression.

SC0062

SC0062 is a highly selective ETA receptor antagonist currently under investigation as a novel therapeutic for IgAN. Endothelin-1 signaling through the ETA receptor contributes to glomerular hypertension, mesangial proliferation, and progressive renal injury, making selective ETA blockade an attractive disease-modifying strategy [14,20]. In a phase 2 randomized, double-blind, placebo-controlled trial, SC0062 achieved a 36.6% reduction in UPCR over 24 weeks compared with placebo (p<0.001), while maintaining stable eGFR and demonstrating a favorable safety profile. Beyond proteinuria reduction, the study reported improvements in hematuria and significant decreases in serum endothelin-1 levels, confirming effective pathway inhibition [35].

Supporting these clinical findings, pharmacokinetic and pharmacodynamic studies in healthy volunteers established SC0062’s dose-proportional exposure, sustained ETA receptor occupancy, and good tolerability, reinforcing its suitability for chronic administration in glomerular diseases [36]. Collectively, these results position SC0062 as a promising candidate for disease modification in IgAN, offering targeted endothelin pathway inhibition that may complement or surpass traditional renin-angiotensin system blockade in reducing proteinuria and preserving kidney function.

Sodium-Glucose Cotransporter 2 (SGLT2) Inhibitors

SGLT2 inhibitors, initially developed for glycemic control in type 2 diabetes, have demonstrated broad renoprotective benefits across diverse chronic kidney disease (CKD) populations, including IgAN. Their mechanism of action involves reducing intraglomerular pressure through modulation of tubuloglomerular feedback, thereby lowering proteinuria and slowing progression of kidney disease independent of glycemic status [14,20]. In the landmark DAPA-CKD trial, dapagliflozin reduced the composite risk of sustained decline in eGFR, ESKD, and renal or cardiovascular death by 39%, with consistent benefit observed in the prespecified subgroup of patients with IgAN. Importantly, these effects were evident regardless of diabetes status, underscoring the renoprotective properties of SGLT2 inhibition beyond glucose lowering [37]. Reflecting this evidence, the KDIGO 2025 Clinical Practice Guideline for Glomerular Diseases recommends SGLT2 inhibitors for patients with IgAN and persistent proteinuria (>0.5-1.0 g/day) despite optimized RAS blockade [16]. Their favorable safety profile, oral administration, and compatibility with both supportive and disease-modifying therapies position SGLT2 inhibitors as an attractive adjunct in the evolving therapeutic landscape of IgAN.

Precision medicine in IgAN

Precision medicine in IgAN is rapidly evolving, shifting the therapeutic paradigm from generalized immunosuppression to individualized, biomarker-driven care. This approach aims to tailor treatment based on a patient’s molecular profile, disease trajectory, and response predictors.

Biomarker-Guided Therapy

Biomarker-guided therapy is transforming the clinical management of IgAN by enabling precision medicine approaches that tailor interventions to individual molecular and immunologic disease signatures. Traditional risk stratification based on proteinuria and eGFR is increasingly complemented by biomarkers that provide mechanistic insight into disease activity and therapeutic responsiveness. Kamal et al. emphasize the utility of Gd-IgA1, anti-Gd-IgA1 autoantibodies, urinary monocyte chemoattractant protein-1 (MCP-1), and complement activation products (C3a, C5b-9) as predictors of disease progression and treatment response, supporting their integration into both diagnostic and therapeutic decision-making. These biomarkers not only facilitate early diagnosis but also help identify patients most likely to benefit from targeted therapies, such as complement inhibitors or B-cell-modulating agents [38].

Expanding this landscape, Xu et al. highlight novel biomarker candidates, including urinary exosomal microRNAs (miRNAs), serum tumor necrosis factor receptors 1 and 2 (TNFR1/2), and proteomic signatures that correlate with histologic severity and long-term outcomes. Such biomarkers provide multidimensional profiling of disease biology, bridging molecular pathways with clinical endpoints [39]. As these tools are increasingly incorporated into clinical trials and practice, biomarker-guided therapy offers a pathway toward precision nephrology, enabling individualized treatment strategies that optimize efficacy, minimize unnecessary immunosuppression, and improve long-term renal outcomes in IgAN.

Risk Stratification

Risk stratification is a cornerstone of modern IgAN management, enabling clinicians to tailor therapeutic strategies and optimize long-term outcomes. The International IgAN Prediction Tool, developed and validated by Barbour et al., integrates both clinical and histopathologic parameters, including age, sex, blood pressure, proteinuria, eGFR, and Oxford-MEST (Mesangial hypercellularity, Endocapillary hypercellularity, Segmental glomerulosclerosis, and Tubular atrophy/interstitial fibrosis) scores, to generate individualized estimates of the risk of progression to kidney failure. This model has demonstrated robust predictive performance across diverse geographic cohorts, reinforcing its utility in global practice and clinical trial design. Importantly, the tool has been instrumental in identifying patients at higher risk who may benefit from early initiation of immunosuppressive or targeted therapies, while sparing low-risk individuals from unnecessary treatment. Recognizing the unique features of pediatric IgAN, the prediction tool was subsequently adapted for children, incorporating age-appropriate parameters while maintaining predictive accuracy, thereby extending its applicability across the disease spectrum [40,41]. These advancements underscore the importance of individualized risk modeling in IgAN and support its integration into routine clinical practice, where precision risk assessment informs both therapeutic decision-making and enrollment criteria for interventional studies.

Combination Therapy

Combination therapy is increasingly recognized as a rational strategy in IgAN, reflecting the disease’s multifactorial pathogenesis that encompasses aberrant mucosal immunity, immune-complex formation, complement activation, and glomerular injury. While monotherapies targeting a single pathway have demonstrated efficacy, they may be insufficient for patients with progressive disease or high-risk features. Cheung et al. emphasize that combining agents with distinct mechanisms of action, such as RAS blockades with angiotensin-converting enzyme inhibitors (ACEIs) or angiotensin receptor blockers (ARBs), immunomodulators like targeted-release budesonide, and complement inhibitors, may provide synergistic benefits by simultaneously reducing proteinuria, modulating immune responses, and attenuating complement-mediated glomerular damage [12]. This multidimensional approach has the potential to optimize efficacy while limiting reliance on broad immunosuppression.

Barratt et al. further highlight the therapeutic potential of dual pathway targeting, particularly combining interventions directed at the lectin pathway of complement activation with upstream immunologic modulation. Such strategies may enhance efficacy by addressing both the drivers of immune-complex formation and the downstream inflammatory cascade, while minimizing toxicity through selective pathway inhibition [42]. As novel agents continue to emerge, rationally designed combinations tailored to individual risk profiles and biomarker signatures, with careful risk-benefit assessment, are poised to redefine the therapeutic landscape of IgAN, moving toward precision medicine approaches that integrate supportive care with disease-modifying therapies.

Clinical guidelines and future directions

The KDIGO 2025 Clinical Practice Guidelines for Glomerular Diseases mark a significant evolution in the management of IgAN, emphasizing early diagnosis, aggressive proteinuria control, and individualized therapy informed by biomarkers and risk stratification [16]. Foundational therapy continues to center on RAS blockade with ACE inhibitors or ARBs, complemented by SGLT2 inhibitors, which have demonstrated robust renoprotective effects across CKD populations. For patients with persistent proteinuria or declining eGFR despite optimized supportive care, the guidelines recommend consideration of targeted agents, including APRIL/BAFF inhibitors and complement blockers, reflecting the growing role of disease-modifying therapies. Importantly, KDIGO highlights the integration of pharmacogenomics to predict drug metabolism and therapeutic response, alongside the use of patient-reported outcomes to capture quality of life and treatment satisfaction, ensuring that patient-centered care complements biological precision [43,44].

## Conclusions

The therapeutic landscape of IgAN is undergoing a paradigm shift from generalized supportive care toward precision, disease-modifying interventions. While ACEIs, ARBs, SGLT2 inhibitors, and dual endothelin/angiotensin receptor antagonists remain foundational therapies, particularly in patients with CKD, emerging agents are increasingly designed to address the underlying immunopathogenesis of IgAN. Targeted approaches such as APRIL and BAFF inhibitors have demonstrated efficacy in reducing pathogenic IgA1 production, while complement blockers mitigate downstream glomerular injury by interrupting key inflammatory cascades.

In parallel, advances in precision medicine tools, including biomarker-guided therapy and validated risk stratification algorithms, are enabling individualized treatment strategies that align therapeutic intensity with patient-specific disease biology and risk profiles. These innovations collectively offer the potential for improved renal outcomes, sustained proteinuria reduction, durable kidney protection, and enhanced patient-centered care. As ongoing trials refine optimal sequencing, duration, and combination strategies, the future of IgAN management lies in personalized, biology-driven care that balances efficacy, safety, and patient preferences, ultimately redefining standards of treatment in glomerular disease.
